# Current status and future directions of multimodal deep learning normal tissue complication probability modelling in head and neck cancer radiotherapy

**DOI:** 10.3389/fonc.2026.1893002

**Published:** 2026-08-28

**Authors:** Victoria Butterworth, Tom Young, Andrew P. King, Teresa Guerrero Urbano

**Affiliations:** 1 School of Cancer & Pharmaceutical Sciences, King’s College London, London, United Kingdom; 2 Radiotherapy, Guy’s and St Thomas’ NHS Foundation Trust, London, United Kingdom; 3 School of Biomedical Engineering & Imaging Sciences, King’s College London, London, United Kingdom

**Keywords:** deep learning, fusion, head and neck, multimodal, NTCP, radiotherapy

## Abstract

Normal tissue complication probability (NTCP) modelling is central to balancing tumour control and toxicity risk in radiotherapy (RT) for head and neck cancer (HNC). Despite major advances in radiation delivery and imaging, toxicity remains a major determinant of long-term quality of life in survivors. Classical NTCP models based on dose–volume histograms (DVHs) and phenomenological equations such as the Lyman–Kutcher–Burman model have provided clinically valuable guidance for decades but oversimplify the complex, multidimensional nature of the normal tissue radiation response. Recent developments in machine learning (ML) and deep learning (DL) have enabled the integration of large-scale clinical, imaging, dosimetric and biological data to generate more accurate, patient-specific toxicity predictions. However, data scarcity, endpoint variability, and limited external generalisability have restricted clinical translation. This mini-review summarises the current status of NTCP modelling in HNC, focusing on evolving multimodal DL approaches and data fusion strategies. A structured literature review approach identified 10 contemporary studies of multimodal DL models for radiotherapy-related toxicity prediction in HNC. Across included studies, fusion architectures were inconsistently reported and model development procedures were also variably described regarding feature selection, hyperparameter tuning, model selection and validation. Current multimodal DL NTCP models are promising but not yet ready for routine clinical use, with robust external validation, calibration assessment, reproducibility, clinical utility evaluation and prospective testing required before integration into RT decision-support workflows.

## 1 Introduction

Advances in radiotherapy (RT) planning and delivery have improved dose conformity in head and neck cancer (HNC), particularly through widespread adoption of intensity-modulated and image-guided RT (1). However, complete sparing of organs at risk (OARs) remains impossible and reducing dose to one structure may inadvertently increase dose to another or compromise tumour control (2). The central challenge of HNC RT therefore remains optimization of the therapeutic ratio: maximising tumour control whilst minimising toxicity (3).

Outcome modelling seeks to quantify these competing probabilities through tumour control probability (TCP) and normal tissue complication probability (NTCP) modelling (4). In HNC, toxicities profoundly affect long-term quality of life. Accurate NTCP models offer the potential to guide patient-centred decision making, inform plan selection and enable risk-adapted follow-up strategies (5–8).

Radiation oncology’s quantitative foundation makes it uniquely positioned to deliver personalised medicine with treatment tailored to patient anatomy, tumour characteristics and individual toxicity susceptibility (9).

### 1.1 Classical NTCP modelling

Classical TCP and NTCP models have provided the foundation for clinical dose-constraint recommendations (10). Traditional analytical models describe empirical relationships between dose and observed outcomes, relying on simplified mathematical representations of biological processes. The linear-quadratic model remains the cornerstone for TCP estimation, whilst the Lyman–Kutcher–Burman (LKB) model remains the most widely used NTCP framework (4). These models typically use OAR dose–volume histogram (DVH) metrics to predict complication rates. The seminal work by Emami et al. (11) and the QUANTEC guidelines (12) established evidence-based tolerance doses which are still reflected in modern protocols.

However, these models carry intrinsic limitations (3). By reducing complex, heterogeneous dose distributions to a few scalar metrics, spatial information within OARs is lost, despite the recognised non-uniformity of both dose deposition and organ radiosensitivity (5) (6). Furthermore, NTCP variation between patients treated to identical OAR doses highlights the influence of additional clinical and biological factors including age, comorbidities, smoking, concurrent chemotherapy, and genetic predisposition (13, 14). Dose distribution accuracy is further affected by segmentation variability and treatment-delivery uncertainties (15, 16).

Although DVH-based NTCP models remain clinically valuable, they cannot capture these multidimensional interactions. More recent modelling efforts have therefore adopted data-driven approaches, leveraging large retrospective datasets to learn patterns predictive of toxicity. These approaches increasingly exploit routinely collected clinical data, imaging features, and dosimetric maps to improve prediction accuracy.

### 1.2 Emergence of machine learning and deep learning approaches

The last decade has witnessed a paradigm shift toward machine learning (ML) and deep learning (DL)-based NTCP modelling (1) (17). Expanding computational capacity and access to electronic health records have enabled integration of diverse datasets encompassing demographics, dosimetry, and imaging biomarkers. Conventional ML approaches including multivariable logistic regression (MVLR), decision trees, random forests, support-vector machines, and Bayesian networks have been widely applied to predict binary toxicity endpoints. MVLR models remain widely used for their interpretability but have limited ability to model complex non-linear relationships, interaction effects and high-dimensional spatial information (18, 19). Feature selection and penalisation methods such as LASSO or ridge regression can reduce overfitting but may still overlook spatially localised or interaction effects relevant to toxicity risk (14).

DL architectures, particularly convolutional neural networks (CNNs), overcome several of these constraints by learning hierarchical spatial features directly from imaging data. RT dose distributions stored as three-dimensional (3D) DICOM dose cubes represent a particularly rich data source because they preserve voxel-level spatial information lost in DVH-based representations (3). CNN-based models can analyse these voxel-level dose maps to learn patterns associated with toxicity outcomes (20).

However, toxicity risk is unlikely to be explained by spatial dose alone, motivating increased interest in multimodal approaches that combine imaging, dosimetric and clinical data within unified prediction frameworks. Following from extensive prior reviews of classical NTCP modelling, radiomics and broader ML approaches (21, 22), this review focuses on multimodal DL as an emerging frontier where learned representations, fusion strategy and validation are central to assessing clinical value.

### 1.3 Multimodal inputs and fusion strategies for NTCP prediction

Radiotherapy toxicity is influenced by interacting anatomical, dosimetric, clinical and biological factors making NTCP prediction inherently multimodal. In this review, multimodal DL specifically refers to models that integrate more than one clinically distinct data domain rather than necessarily distinct machine-learning input modalities, including clinical, imaging and dosimetric data. Clinical data are typically represented as structured tabular variables whereas imaging and dosimetric data may be represented as spatial inputs. When multiple data domains are available, a key modelling decision is how they should be combined to improve predictive power (23, 24).

Fusion strategies are typically categorised as early, intermediate and late according to the stage at which information from different input domains is combined (23). In early fusion, raw inputs or pre-extracted features (including handcrafted features or fixed CNN-derived representations) are concatenated before shared model learning. In intermediate (joint) fusion, modality-specific neural network branches learn separate feature representations before combination into a final model. Because the model is trained end-to-end, loss can be backpropagated to the feature extracting branches, enabling representations to be optimised for the prediction task. In late fusion, predictions from modality-specific models are aggregated at the decision level e.g. through weighted voting or averaging.

## 2 Current applications in HNC

### 2.1 CNN-based spatial dose and imaging models

Contemporary multimodal DL studies in HNC NTCP modelling have mostly focused on incorporating spatial dose and anatomical imaging information into CNN-based toxicity prediction frameworks. Early work by Men et al. (25) used a 3D regional CNN combining planning CT, dose distribution and salivary gland segmentations to predict grade ≥2 xerostomia by 12 months, demonstrating improved discrimination compared with conventional multivariable approaches. Subsequent work by Chu et al. (26) extended this concept in a larger cohort with independent and external validation using deep CNN architectures including EfficientNetV2-S and ResNet and incorporating clinical data for xerostomia prediction at 12 months. Their attention maps identified regions posterior to the mandible within the parotid glands, corresponding to biologically plausible salivary duct and stem-cell-rich regions previously implicated in xerostomia risk (27). However, external validation performance was lower than internal validation performance and a reference NTCP model outperformed the DL models in the external cohort, highlighting that biologically plausible learned features do not necessarily guarantee immediately transferrable robust cross-institutional performance. Transfer learning using DL models initialised with pretrained model weights obtained from the internal training data increased external cohort performance beyond that of the reference NTCP model, suggesting that DL-based NTCP models may require local adaption before clinical deployment.

### 2.2 Transfer learning and limited data strategies

Transfer learning has also been proposed to counter limited sample sizes (28) with Dohopolski et al. (29) finding improved performance in predicting feeding tube need or weight loss from CT, cone-beam CT and dose cubes using a pretrained ResNet-50 model, MedicalNet, compared to training *de novo*. Nevertheless, transfer learning remains less established for RT-specific inputs such as dose cubes than for conventional diagnostic imaging. To supplement DL on dose cubes, some studies used handcrafted dosiomic features in addition to DL with Xiang et al. (30) demonstrating increased predictive ability for radiation dermatitis in nasopharyngeal patients combining dosiomic and DL features from a ResNet-34 model. ResNet models have been used on a variety of input modalities beyond conventional imaging including serial CBCT-derived Jacobian matrices for predicting feeding tube, hospitalisation and radionecrosis by Bang et al. (31) and clinical data, dose distributions, CT and organ at risk segmentations for prediction of dysphagia by De Vette et al. (32). Lin et al. (33) went beyond using only OAR segmentations for NTCP modelling and found enhanced performance in predicting xerostomia when tumour (GTVp) segmentation was incorporated alongside parotid gland models through decision-level fusion.

### 2.3 Fusion strategy comparisons

Several studies have directly explored how multimodal information should be combined. Humbert-Vidan et al. (34) compared early, intermediate and late fusion strategies for osteoradionecrosis prediction using clinical variables and mandibular dose maps, reporting superior discrimination and calibration for late fusion while intermediate fusion achieved a more balanced distribution of probabilities. De Vette et al. (32) explored integration points of combining their dose, CT and segmentation ResNet-3D model with the clinical data 1D-ResNet model, finding intermediate fusion performed best on independent and external testing. Most studies used a single fusion methodology with few acknowledging that multimodal fusion strategy can materially affect model behaviour, but that also optimal fusion may be endpoint, dataset and architecture specific (29, 35).

### 2.4 Multi-toxicity modelling and comparative performance

More recent work has begun to move beyond single-endpoint prediction towards broader toxicity representations. MacRae et al. (36) developed a multi-toxicity DL model to jointly predict aspiration, dysphagia, sticky saliva, taste alteration and xerostomia at 6 months using CT, dose, OAR segmentations and clinical variables within a jointly optimised multitask framework. The multi-toxicity model improved prediction for some endpoints including dysphagia and xerostomia but not others with single-toxicity or conventional clinical NTCP models remaining competitive for sticky saliva, taste alteration and aspiration. Similarly, Bang et al. (31) were unable to demonstrate imaging added significant predictive power compared to a clinical variable model alone and Men et al. (25) acknowledged their DL model produced similar performance to existing LR models using clinical and DVH parameters with hand-crafted biomarkers from CT and MR. Conversely, De Vette et al. (32) and Dohopolski et al. (29) both demonstrated improved performance with DL models over clinical models whilst Chu et al. (26) demonstrated improved performance with DL on an internal cohort but found this was not generalisable to an external cohort. Leave-one-out analyses also suggested that dose information may be particularly important with removal of dose cubes leading to larger performance reductions than removal of CT or segmentation inputs in several studies (33, 36).

### 2.5 Study heterogeneity, validation and reporting

**Figure 1:**
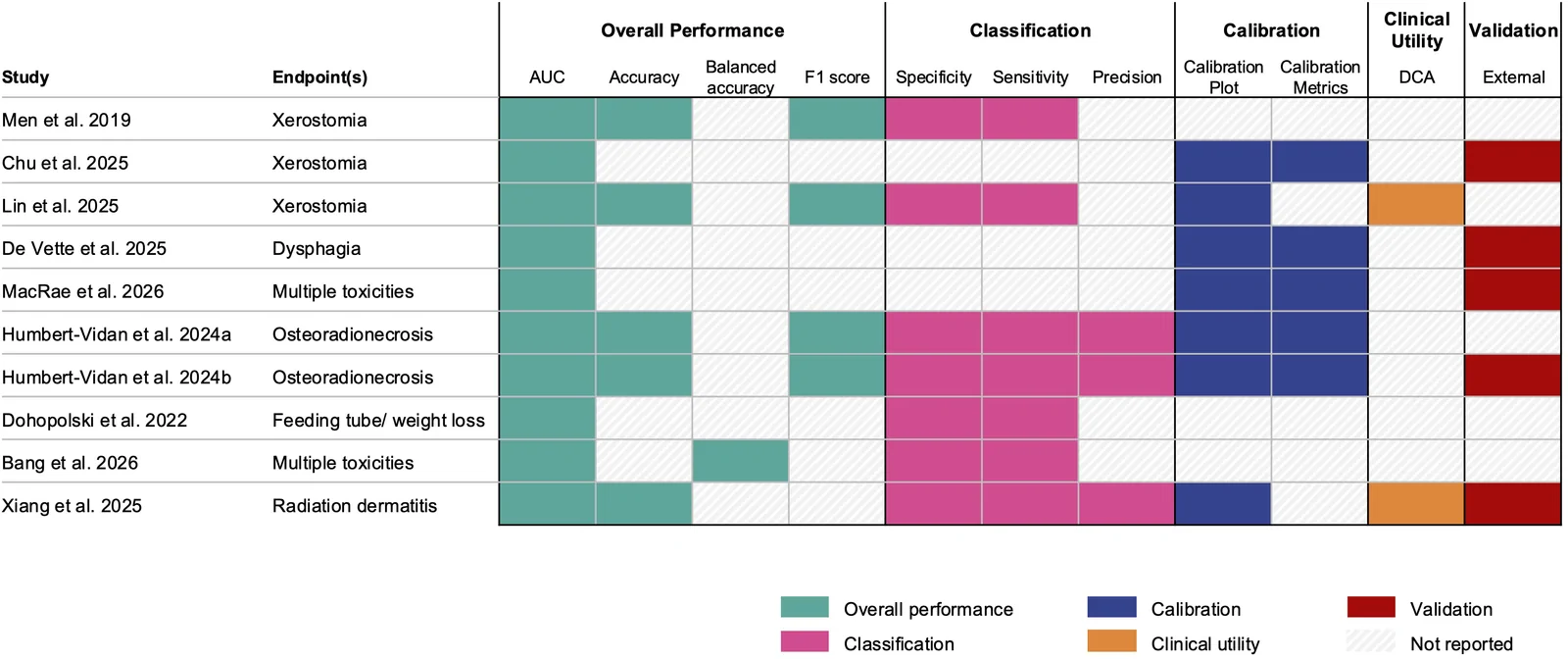
Evaluation and reporting metrics in multimodal deep learning NTCP studies for head and neck cancer. DCA, Decision Curve Analysis, Quantitative calibration metrics includes Brier score, Log Loss, Nagelkerke’s R^2^, Adapted Calibration Error and Expected Calibration Error.

Overall, current applications demonstrate that multimodal DL can integrate heterogeneous RT data, including spatial dose information, anatomical imaging, clinical variables, longitudinal treatment imaging and multiple correlated toxicity endpoints. However, performance gains over clinical or established NTCP models are inconsistent, particularly in external validation studies, and none have demonstrated robust performance sufficient for clinical translation. The included studies also vary substantially in toxicity endpoint, input modalities, fusion strategies and validation design. Key characteristics of identified multimodal DL NTCP studies in HNC are summarised in **Table 1**. Fusion type was determined through examining network diagrams and descriptions within the text and because several studies used more than one fusion operation, **Table 1** reports the final multimodal fusion strategy, defined as the stage at which all included data domains were integrated into the predictive architecture. For example, models which combined CT, dose and segmentation at input but used intermediate fusion to add clinical data were classified as intermediate fusion. **Supplementary Table 1** extends **Table 1** to describe event numbers, training and validation strategies per model. **Figure 1** displays the heterogeneity of reported metrics across the identified studies.

**Table 1:** Identified published studies investigating deep learning multimodal head and neck cancer normal tissue complication probability modelling.

Study	Endpoint	Input data domains	Cohort size			DL architecture & model details	Final multimodal fusion strategy	Main findings and reported discrimination
			Development	Internal test/CV	External			
Men et al., 2019 (25)	Xerostomia at 12 months post RT	I, D	706	78	–	3D residual CNN	Intermediate	DL showed higher internal discrimination than clinical logistic regression: AUC 0.84 (95% CI 0.74–0.91) vs 0.74 (95% CI 0.64–0.84).
Chu et al., 2025 (26)	Xerostomia at 12 months post RT	C, I, D	759	138	311	CNN, ResNet, EfficientNetV2-S	Intermediate	DL outperformed reference NTCP internally but not externally. Transfer learning moderately improved external performance vs *de novo* training: AUC 0.66 (95% CI 0.63–0.69) vs 0.63 (95% CI 0.60–0.66).
Lin et al., 2025 (33)	Xerostomia at 6 months	I, D	137	43	–	3D-CNN	Late	DL models showed higher reported discrimination than handcrafted radiomics/dosiomics ML models: AUC 0.86 vs 0.71. Combined GTVp and parotid-based networks achieved higher AUC than either network alone: 0.86 vs 0.75 and 0.81, respectively.
De Vette et al., 2025 (32)	Dysphagia at 6 months post-RT	C, I, D	941	171	338	ResNet (3D & 1D)	Early, intermediate, late	DL models showed higher discrimination than conventional NTCP models in both internal and external validation: internal AUC 0.84 (95% CI 0.77–0.90) vs 0.76 (95% CI 0.68–0.84); external AUC 0.74 (95% CI 0.68–0.80) vs 0.63 (95% CI 0.56–0.69). Intermediate fusion showed best calibration.
MacRae et al., 2026 (36)	Aspiration, dysphagia, sticky saliva, taste alteration, xerostomia at 6 months.	C, I, D	872	218	328	3D-DenseNet-121 + Vision Transformer + Fully Connected Network	Intermediate	Multitoxicity models showed endpoint-dependent benefit. In external validation, multitoxicity DL improved dysphagia prediction compared with single-endpoint DL and logistic regression: AUC 0.71 (95% CI 0.64–0.77) vs 0.66 (95% CI 0.58–0.72) and 0.67 (95% CI 0.60–0.73), respectively. For aspiration, logistic regression performed best: AUC 0.72 (95% CI 0.61–0.83) vs 0.68 for both multitoxicity DL (95% CI 0.52–0.81) and single-endpoint DL (95% CI 0.54–0.81).
Humbert-Vidan et al., 2024a (34)	Osteoradionecrosis	C, D	184	Nested 5-fold CV	–	3D-DenseNet-40	Early, intermediate, late	Late fusion showed the highest reported discrimination and calibration, although AUCs were similar across fusion strategies: late AUC 0.70 (95% CI 0.63–0.78), early AUC 0.69 (95% CI 0.62–0.77), joint AUC 0.68 (95% CI 0.61–0.76). Joint fusion produced more balanced predicted probabilities.
Humbert-Vidan et al., 2024b (35)	Osteoradionecrosis	C, D	184	Nested 5-fold CV	82	3D-DenseNet-40	Intermediate	No significant difference was observed between DL and logistic regression models, or between internal and external validation performance; external AUC was 0.66 (95% CI 0.54–0.78) for DL versus 0.64 (95% CI 0.54–0.74) for logistic regression.
Dohopolski et al., 2022 (29)	Feeding tube placement or >10% weight loss during RT	C, I, D	271	CV	–	MedicalNet & LR	Late	DL model incorporating imaging and dose improved discrimination over the clinical model alone: AUC 0.75 (95% CI 0.69–0.81) vs 0.69 (95% CI 0.63–0.76).
Bang et al., 2026 (31)	Feeding tube placement, hospitalisation within 4 months treatment. RN within 2 years.	C, I, D	1,012	CV	–	ResNet + MLP	Intermediate	Adding dosimetric or imaging data did not improve prediction over clinical features across endpoints; for feeding-tube prediction, AUC was 0.721 ± 0.020 for clinical + weight loss + deformation matrix features vs 0.745 ± 0.034 for clinical + weight loss features.
Xiang et al., 2025 (30)	Radiation dermatitis (up to 2 years post-treatment)	C, D	167	72	51	ResNet34, DenseNet-121, MobileNet + MLP	Intermediate	DL + dosiomics + clinical features showed marginally higher external validation performance than DL alone: AUC 0.82 (95% CI 0.67–0.95) vs 0.81 (95% CI 0.68–0.92).

Development cohort refers to the dataset used for model training and where applicable, hyperparameter tuning or model selection. Internal test/CV refers to performance assessment within the same source population, using either a held-out internal test set, cross-validation or nested cross-validation in which the inner folds were used for hyperparameter tuning and performance was estimated using held-out outer folds. Fusion strategy refers to the final stage at which all included data domains were integrated in the predictive architecture. For studies evaluating multiple fusion strategies, each reported strategy is listed. AUC values are reported as presented by study authors. CI, Confidence Interval; C, Clinical tabular data (including DVH metrics); I, Imaging Data (3D tensor); D, Dose (3D tensor); CV, cross-validation; NR, Not Reported; RN, Radionecrosis; LR, Logistic Regression; DL, Deep Learning; MLP, Multilayer Perceptron.

## 3 Discussion

Despite rapid methodological advances, NTCP models—particularly in HNC—remain largely research tools rather than routine clinical aids (3). Multimodal DL can potentially improve prediction in some settings, but its added value is endpoint, dataset and validation dependent rather than universal with barriers spanning the entire modelling pipeline.

### 3.1 Data limitations and generalisability

Many multimodal NTCP studies in HNC rely on relatively small, retrospective single-centre datasets with variable follow-up duration and inconsistent endpoints. This creates a substantial risk of overfitting, particularly in high-dimensional settings where the number of candidate features may approach or exceed the number of outcome events. Although feature-selection and dimensionality-reduction strategies may partially mitigate these effects, they may also discard subtle multimodal interactions relevant to toxicity prediction. Furthermore, multimodal DL models are inherently data intensive and may require substantially larger datasets than conventional clinical prediction models to achieve robust and generalisable performance. Many current models are therefore likely underpowered, particularly when high-dimensional imaging or dose inputs are trained on relatively small cohorts, especially those with few toxicity events (see **Supplementary Table 1**), increasing the risk of overfitting and optimistic internal validation.

The development of large multimodal RT datasets requires considerable technical, financial and governance infrastructure including image curation, harmonisation, linkage of longitudinal clinical outcomes and secure data-sharing frameworks (37–39). Consequently, the contemporary multimodal DL NTCP literature remains concentrated within a relatively small number of research groups and institutions. Several identified studies originated from overlapping institutional datasets, raising concerns regarding limited independent replication and restricted population diversity despite nominally multicentre designs. While such concentration reflects the practical challenges of multimodal AI research, it may reduce confidence in the external generalisability of published models across broader, more global healthcare settings.

Collaborative prospective initiatives such as *Hamlet.rt* (40) and PREDMORN (41) may help address some of these limitations through harmonised multicentre data collection. Greater demographic, socioeconomic and treatment heterogeneity will be essential to ensure equitable model performance across patient subgroups. However, subgroup analyses and fairness assessments remain uncommon within the contemporary multimodal DL literature despite increasing emphasis within AI reporting guidelines such as TRIPOD-AI (42).

Integration of biological and genetic data is currently absent in the contemporary multimodal DL literature. The normal tissue radiation response may be influenced by heritable radiosensitivity traits, although the estimated contribution of genetic factors varies by endpoint, tissue type, toxicity scoring system and study population (43, 44). Genome-wide association studies have linked single-nucleotide polymorphisms with RT toxicity in HNC suggesting that biological data may provide complementary information to clinical, imaging and dosimetric predictors (45–48). Deneuve et al. combined a radiosensitivity blood assay with dosimetric parameters to predict mucositis (49); however no published study has yet achieved comprehensive multimodal fusion across clinical, imaging, dosimetric and biological domains. Incorporating biological data into NTCP modelling additionally remains challenging because of limited data availability, cost, assay heterogeneity, privacy considerations and regulatory requirements. As molecular and functional radiosensitivity data become more accessible and collaborations across domain experts grow, carefully validated multimodality modelling frameworks will clarify if these inputs improve patient-specific toxicity prediction (8).

### 3.2 Endpoint heterogeneity

NTCP modelling is hampered by the variety of toxicity scoring systems (CTCAE, EORTC, MDASI-HN and PSS-HN, RTOG, LENT-SOMA) and by variable and potentially arbitrary reporting timepoints. External validation of models requires assumptions of scoring system equivalency, adding an additional challenge for generalisability. Physician-reported scales often underestimate patient-reported symptoms, while dichotomisation of ordinal outcomes reduces granularity, limiting future clinical applicability, and may create unstable labels when toxicity grading is subjective. Longitudinal modelling that captures the temporal evolution of toxicity is needed to reflect real-world patient experience. Moreover, many patients experience multiple correlated toxicities (50) suggesting that focus should be directed to predict joint or composite interrelated endpoints rather than single toxicities in future models.

### 3.3 Reporting, interpretability and reproducibility

More complex architectures such as ensemble models and DL networks may achieve higher accuracy but often at the expense of transparency compared with conventional clinical prediction models (18). Explainability techniques including attention maps and SHAP values can help clinicians interrogate model behaviour, fostering trust in cases where predictions are driven by clinically plausible regions or features. However, explainability outputs should not be interpreted as proof of causal relevance and visually plausible explanations may still reflect confounding or dataset bias and thus also require careful validation before being used to support clinical trust (51).

Reporting quality remains a major limitation within the current literature. As **Figure 1** demonstrates, whilst all studies reported discrimination metrics such as AUC, other aspects of model development and evaluation were inconsistently presented, especially handling of missing data, hyperparameter tuning and calibration, limiting comparison across studies. Greater adherence to reporting guidance such as TRIPOD-AI and CLAIM alongside risk-of-bias assessment using PROBAST-AI would improve transparency and reproducibility (42, 52, 53).

A particular issue identified in this review was inconsistent reporting of multimodal fusion architectures. In several studies, it was challenging to determine where modalities were combined, whether modality-specific encoders were jointly optimised and how clinical variables were integrated with imaging or dosimetric representations. Reporting was also unclear regarding whether feature selection, dimensionality reduction or other methods were used to control the size of fused feature vectors. Few studies explicitly justified their chosen fusion strategy, despite evidence that early, intermediate and late fusion approaches may produce different behaviour and calibration (23, 32, 34). Additionally, model development procedures were sometimes incomplete, while hyperparameter tuning, model selection and validation were not always clearly separated, potentially resulting in biased performance estimates.

Finally, source code and trained model availability were limited across the reviewed literature. In some cases, code was reported as available, but corresponding repository links were inaccessible or non-functional at time of review. Whilst clinical data sharing may be limited by governance and privacy constraints, publication of code, model configuration files, preprocessing pipelines and trained weights where appropriate would substantially improve reproducibility. Reporting guidelines and journals both require or encourage authors to publish the code and models developed for AI research in repositories with permanent identifiers (42, 54).

### 3.4 Clinical translation

Ultimately, for NTCP models to have clinical value, they should be embedded within decision-support systems that guide RT planning and adaptation, a consideration not explicitly accounted for across the contemporary literature. Integration with treatment-planning software could allow real-time toxicity risk estimation for candidate plans, supporting trade-offs between tumour coverage and toxicity. These tools should undergo prospective evaluation analogous to any clinical intervention (42, 55). The ongoing *Pre-ACT* study, which tests whether providing ML-based toxicity-risk information influences outcomes, is an example of such translational research (55).

To achieve clinical adoption, NTCP models must demonstrate not only strong internal performance, but also robust external validation across diverse populations, institutionsand treatment practices in addition to interpretability and workflow compatibility. For clinical NTCP use, discrimination alone is insufficient; models must provide reliable absolute risk estimates through calibration assessment and demonstrate clinical utility through approaches such as decision curve analysis, analysis notably missing in the majority of the published studies. This is particularly important where predictions may influence treatment planning choices, patient counselling or intervention. Uncertainty quantification approaches, including Monte Carlo drop out and deep ensembles may further support confidence-aware prediction and selective clinical rollout (56). Regulatory approval, clinician education, and bias mitigation will be crucial (57–59). When validated and deployed responsibly, multimodal AI decision-support tools hold promise to improve HNC RT outcomes through personalised risk prediction and adaptive planning (60).

## 4 Conclusions and future directions

NTCP modelling in HNC has progressed from simple DVH-based analytical equations to sophisticated multimodal DL frameworks. While classical models such as LKB remain integral to clinical planning, modern data-driven methods incorporating clinical, imaging, and biological inputs are revealing the multidimensional complexity of normal tissue responses.

The contemporary literature reveals that integrating spatial dose information and multimodal features inconsistently improve predictive power and introduce new challenges of data scarcity, endpoint heterogeneity, and generalisability. Current multimodal DL NTCP models remain promising but are not yet ready for routine clinical use. Progress will require larger standardised multicentre datasets, harmonised toxicity endpoints, transparent model development, robust external validation, calibration assessment, reproducibility, clinical utility evaluation and prospective testing before integration into RT decision-support workflows. If these challenges are addressed, next-generation NTCP models have the potential to enable personalised RT planning that may optimise tumour control while minimising toxicity, ultimately improving long-term quality of life for HNC patients.

## 5 Methods

### 5.1 Protocol and registration

The article was conducted as a structured mini-review rather than a formal systematic or scoping review. No formally registered protocol was registered. The methods were developed by the review team and documented before screening commenced, adhering to the Preferred Reporting Items for Systematic reviews and Meta-Analyses extension for Scoping Reviews (PRISMA-ScR) statement (61) to improve transparency in study identification and selection.

### 5.2 Eligibility criteria

Studies were considered eligible for inclusion if they included human patients with HNC treated with radiotherapy and reported a multimodal DL model for prediction of radiotherapy-related toxicity or NTCP outcomes. Studies were eligible if the predictive framework incorporated DL architectures that learned feature representations from one or more imaging, dosimetric or other high-dimensional input modalities within a multimodal toxicity prediction framework. Multimodal was defined as the integration of two or more distinct data domains within the predictive model, such as clinical, imaging, radiomic, dosimetric, pathological or genomic data. Multiple variables derived from a single domain were not considered multimodal. Models using only tabular clinical variables and DVH metrics were considered multimodal only if they additionally incorporated learned representations from high-dimensional imaging, dosimetric or spatial input.

Studies were excluded if they concerned tumours of the oesophagus, thyroid or skin of the head and neck region or if they were review studies, meta-analyses, conference abstracts, duplicate studies or self-published without peer review. Additionally, studies were excluded where DL was used only as an upstream tool such as autosegmentation to create regions of interest, image pre-processing and feature generation for a later non-DL model unless the final predictive model itself was also a multimodal DL model.

The search was restricted to studies published from 2016 onwards to reflect the contemporary era of DL. Restriction to English language was applied for feasibility. The focus on multimodal DL was chosen to enable in-depth examination of fusion architectures and related methodological issues rather than the broader HNC NTCP literature which has already been well documented elsewhere (21, 22).

### 5.3 Information sources

Electronic searches were conducted in PubMed, Ovid MEDLINE, and Ovid Embase on 17/03/2026. The search strategy combined controlled vocabulary and free-text terms to build a five-block search composed of HNC, radiotherapy, toxicity or NTCP outcomes, DL and prediction modelling. Additional records were identified through citation searching of relevant review articles and included studies. The full search strategy for PubMed is shown in Appendix 1. Equivalent search strategies were translated for Ovid MEDLINE and Ovid Embase using database appropriate syntax and controlled vocabulary.

### 5.4 Selection of sources of evidence

Search results were exported to Covidence (Veritas Health Innovation, Melbourne, Australia) for de-duplications and screening. Title/abstract and full-text screening were undertaken by a single reviewer (VB). A PRISMA-style flow diagram summarising study selection is shown in **Supplementary Material Figure 1**.

### 5.5 Data charting, data items and appraisal and synthesis of results

Data was extracted using a standardised extraction form developed by the review team. The charting form was designed to capture key study characteristics and methodological details relevant to the review objectives with particular focus on multimodal fusion approaches. Variables sought included study identification, study design, population and treatment characteristics, toxicity endpoint, input modalities, DL model type, multimodal fusion approach, validation strategy, reported performance measures. Simple counts were used where appropriate, and a narrative synthesis was undertaken to identify common patterns and evidence gaps. Study authors were not contacted for further information.

### 5.6 Review scope and methodological considerations

This review was conducted as a structured mini-review rather than a formal systematic or scoping review. Screening and data extraction were performed by a single reviewer, which may have introduced selection or extraction bias, and no formal quality or risk-of-bias assessment was undertaken. Although reporting, validation, calibration and reproducibility were discussed with reference to TRIPOD-AI, CLAIM and PROBAST-AI guidance, included studies were not formally scored using these tools. Despite these limitations, the review highlights clear priorities for future multimodal NTCP modelling including larger multi-institutional datasets, harmonised toxicity endpoints, transparent reporting of model development and rigorous ongoing validation.
