# Supplementary material for: Current status and future directions of multimodal deep learning normal tissue complication probability modelling in head and neck cancer radiotherapy
Source: Front Oncol. 2026 Aug 28;16:1893002. doi: 10.3389/fonc.2026.1893002 (PMC13563208; doi:10.3389/fonc.2026.1893002)
Supplement: Supplementary file 1 [file Table1.docx]

**Supplementary Material**

**Supplementary Tables**

| **Study** | **Toxicity** | **Development events/total cohort (%)** | **Internal validation events/total cohort (%)** | **External validation events/total cohort (%)** | **Event, training & validation details** |
| --- | --- | --- | --- | --- | --- |
| Men et al. 2019 (1) | Xerostomia | NR/706 | NR/78 | - | Random test-set, event count per split not reported (279 total), cases in toxicity class oversampled during training. |
| Chu et al. 2025 (2) | Xerostomia | 269/759 (35) | 49/138 (36) | 80/311 (26) | 10-fold stratified cross-validation, ensemble predictions for test sets. |
| Lin et al. 2025 (3) | Xerostomia | 80/137 (58) | 24/43 (56) | - | Hold out validation approach with stratified sampling, temporally separated test set |
| De Vette et al. 2025 (4) | Dysphagia | 233/941 (25) | 45/171 (26) | 78/338 (23) | 5-fold CV used to select hyperparameters, ensemble predictions for test sets. |
| MacRae et al. 2026 (5) | Aspiration | 59/872 (7) | 19/218 (9) | 16/328 (5) | 5-fold CV stratified by RT technique, tumour location, CT contrast and CT metal artefact rating. |
|  | Dysphagia | 210/872 (24) | 48/218 (22) | 80/328 (24) |  |
|  | Sticky saliva | 260/872 (30) | 61/218 (28) | 67/328 (20) |  |
|  | Taste alteration | 202/872 (23) | 52/218 (24) | 114/328 (35) |  |
|  | Xerostomia | 334/872 (38) | 93/218 (43) | 128/328 (39) |  |
| Humbert-Vidan et al. 2024a (6) | Osteoradionecrosis | 92/184 (50) | 92/184** (50) | - | Stratified nested 5-fold cross-validation |
| Humbert-Vidan et al. 2024b (7) | Osteoradionecrosis | 92/184 (50) | 92/184** (50) | 41/82 (50) | Stratified nested 5-fold cross-validation. |
| Dohopolski et al. 2022 (8) | Feeding tube placement or >10% weight loss during RT | 115/271 (42) | 115/271* (42) | - | 5-fold CV stratified by toxicity distribution with class-weighted loss. |
| Bang et al. 2026 (9) | Feeding tube placement | 168/1012 (17) | 168/1012* (17) | - | 5-fold CV stratified by toxicity distribution with class-weighted loss. |
|  | Hospitalisation | 43/1012 (4) | 43/1012* (4) | - |  |
|  | Radionecrosis | 47/1012 (5) | 47/1012* (5) | - |  |
| Xiang et al. 2025 (10) | Radiation dermatitis | 76/167 (46) | 32/72 (44) | 26/51 (51) | 5-fold CV applied during feature selection to avoid overfitting |

Supplementary Table 1: Identified published studies investigating deep learning multimodal head and neck cancer normal tissue complication probability modelling. Abbreviations: CV= cross-validation, NR= Not Reported, *cross-validation, **stratified nested 5-fold cross-validation

**Appendix 1:** **Example of literature search:** PubMed Literature search (n=211)

( "Head and Neck Neoplasms"[Mesh] OR (("head and neck"[tiab]) AND (cancer*[tiab] OR neoplasm*[tiab] OR tumor*[tiab] OR tumour*[tiab] OR carcinoma*[tiab] OR malignan*[tiab])) OR HNSCC[tiab] OR "head and neck squamous cell carcinoma"[tiab] OR ((head and neck[tiab] OR oropharyn*[tiab] OR laryn*[tiab] OR hypopharyn*[tiab] OR nasopharyn*[tiab] OR "oral cavity"[tiab] OR tongue[tiab] OR tonsil*[tiab] OR salivary[tiab] OR parotid[tiab]) AND (cancer*[tiab] OR carcinoma*[tiab] OR neoplasm*[tiab] OR tumor*[tiab] OR tumour*[tiab])) ) AND ( "Radiotherapy"[Mesh] OR "Radiotherapy, Intensity-Modulated"[Mesh] OR "Proton Therapy"[Mesh] OR radiotherap*[tiab] OR "radiation therap*"[tiab] OR irradiation[tiab] OR IMRT[tiab] OR VMAT[tiab] OR "intensity modulated"[tiab] OR "volumetric modulated"[tiab] OR proton*[tiab] OR chemoradiotherap*[tiab] OR "chemo-radiotherap*"[tiab] OR CRT[tiab] OR RT[tiab] ) AND ( "Xerostomia"[Mesh] OR xerostomia[tiab] OR dysphagia[tiab] OR "Mucositis"[Mesh] OR mucositis[tiab] OR "Fibrosis"[Mesh] OR fibrosis[tiab] OR "Osteoradionecrosis"[Mesh] OR osteoradionecrosis[tiab] OR ORN[tiab] OR "Trismus"[Mesh] OR trismus[tiab] OR "Dermatitis"[Mesh] OR dermatitis[tiab] OR "normal tissue complication probability"[tiab] OR NTCP[tiab] OR "normal tissue complication*"[tiab] OR toxic*[tiab] OR complication*[tiab] OR "adverse effect*"[tiab] OR "late effect*"[tiab] OR "side effect*"[tiab] OR "functional outcome*"[tiab] OR morbidity[tiab] OR "treatment related"[tiab] OR "feeding tube"[tiab] OR "tube feeding"[tiab] OR aspiration[tiab] OR swallow*[tiab] OR "radiotherapy toxicity"[tiab] OR "treatment complication"[tiab] OR "salivary dysfunction"[tiab] ) AND ( "Deep Learning"[Mesh] OR "Neural Networks, Computer"[Mesh] OR "deep learning"[tiab] OR "deep neural network*"[tiab] OR "neural network*"[tiab] OR CNN[tiab] OR CNNs[tiab] OR "convolutional neural"[tiab] OR transformer*[tiab] OR autoencoder*[tiab] OR multimodal[tiab] OR "multi-modal"[tiab] OR fusion[tiab] OR "data fusion"[tiab] OR "multi-input"[tiab] OR "multi-branch"[tiab] ) AND ( predict*[tiab] OR model*[tiab] OR prognos*[tiab] OR risk[tiab] ) AND ("2016/01/01"[pdat] : "3000"[pdat]) AND english[lang] NOT preprint[pt]

**Supplementary Figures**

**Supplementary Figure 1: PRISMA-style flow diagram showing literature attrition at study identification, screening and selection.**

**References**

1.	Men K, Geng H, Zhong H, Fan Y, Lin A, Xiao Y. A Deep Learning Model for Predicting Xerostomia Due to Radiation Therapy for Head and Neck Squamous Cell Carcinoma in the RTOG 0522 Clinical Trial. *International Journal of Radiation Oncology*Biology*Physics* (2019) 105:440–447. doi: 10.1016/j.ijrobp.2019.06.009

2.	Chu H, de Vette SPM, Neh H, Sijtsema NM, Steenbakkers RJHM, Moreno A, Langendijk JA, van Ooijen PMA, Fuller CD, van Dijk L V. Three-Dimensional Deep Learning Normal Tissue Complication Probability Model to Predict Late Xerostomia in Patients With Head and Neck Cancer. *International Journal of Radiation Oncology*Biology*Physics* (2025) 121:269–280. doi: 10.1016/j.ijrobp.2024.07.2334

3.	Lin L, Ren Y, Jian W, Yang G, Zhang B, Zhu L, Zhao W, Meng H, Wang X, He Q. A deep learning model for predicting radiation-induced xerostomia in patients with head and neck cancer based on multi-channel fusion. *BMC Med Imaging* (2025) 25:305. doi: 10.1186/s12880-025-01848-1

4.	de Vette SPM, Neh H, van der Hoek L, MacRae DC, Chu H, Gawryszuk A, Steenbakkers RJHM, van Ooijen PMA, Fuller CD, Hutcheson KA, et al. Deep learning NTCP model for late dysphagia after radiotherapy for head and neck cancer patients based on 3D dose, CT and segmentations. *Radiotherapy and Oncology* (2025) 213:111169. doi: 10.1016/j.radonc.2025.111169

5.	MacRae DC, van der Hoek L, de Vette SPM, Neh H, Moreno AC, Fuller CD, Langendijk JA, Valdenegro-Toro MA, Sijtsema NM, van Ooijen PMA, et al. A multi-toxicity deep learning approach for normal tissue complication probability modelling in head and neck cancer patients receiving radiotherapy. *Radiotherapy and Oncology* (2026) 219:111486. doi: 10.1016/j.radonc.2026.111486

6.	Humbert-Vidan L, Patel V, King AP, Urbano TG. Comparison of deep-learning multimodality data fusion strategies in mandibular osteoradionecrosis NTCP modelling using clinical variables and radiation dose distribution volumes. *Phys Med Biol* (2024) 69:20NT01. doi: 10.1088/1361-6560/ad8290

7.	Humbert-Vidan L, Hansen CR, Patel V, Johansen J, King AP, Guerrero Urbano T. External validation of a multimodality deep-learning normal tissue complication probability model for mandibular osteoradionecrosis trained on 3D radiation distribution maps and clinical variables. *Phys Imaging Radiat Oncol* (2024) 32:100668. doi: 10.1016/j.phro.2024.100668

8.	Dohopolski M, Wang K, Morgan H, Sher D, Wang J. Use of deep learning to predict the need for aggressive nutritional supplementation during head and neck radiotherapy. *Radiotherapy and Oncology* (2022) 171:129–138. doi: 10.1016/j.radonc.2022.04.016

9.	Bang C, Gautier H, Le WT, Lalonde A, Bernard G, Markel D, Nguyen-Tan F, Filion E, O’Sullivan B, Ayad T, et al. Dynamic prediction of Radiotherapy toxicities in Head and neck cancer using clinical and imaging data. *Radiotherapy and Oncology* (2026) 214:111312. doi: 10.1016/j.radonc.2025.111312

10.	Xiang Z, Yu X, Lin S, Wang D, Huang W, Fu W, Zhu X, Shao L, Wu J, Zheng Q, et al. Deep learning dosiomics for the pretreatment prediction of radiation dermatitis in nasopharyngeal carcinoma patients treated with radiotherapy. *Radiotherapy and Oncology* (2025) 209:110951. doi: 10.1016/j.radonc.2025.110951
